# St. John’s Wort Regulates Proliferation and Apoptosis in MCF-7 Human Breast Cancer Cells by Inhibiting AMPK/mTOR and Activating the Mitochondrial Pathway

**DOI:** 10.3390/ijms19040966

**Published:** 2018-03-23

**Authors:** Mi-Kyoung You, Hwa-Jin Kim, Ji Hyun Kook, Hyeon-A Kim

**Affiliations:** 1 Department of Nutrition and Health Sciences, University of Nebraska-Lincoln, Lincoln, NE 68583, USA; myou3@unl.edu; 2 Hisol Inc., 247-9, Baraebong-gil, Unbong-eup, Namwon-si, Jeollabuk-do 55717, Korea; tailove2212@naver.com; 3 Department of Food and Nutrition, Mokpo National University, 1666, Yeongsan-ro, Cheonggye-myeon, Muan-gun, Jeollanam-do 58554, Korea; kookji76@daum.net

**Keywords:** St. John’s Wort, breast cancer, growth inhibition, AMPK/mTOR, mitochondrial pathway

## Abstract

St. John’s Wort (SJW) has been used as an estrogen agonist in the systems affected by menopause. Also, hypericin, a bioactive compound of SJW, has been used as a photosensitizer in photodynamic therapy. In the present study, we investigate the anti-proliferative and pro-apoptotic effects of SJW to demonstrate the chemo-preventive effect in human breast cancer cells. MCF-7 cells were cultured with DMSO or various concentrations of SJW ethanol extract (SJWE). Cell viability, proliferation, apoptosis, the expression of proteins involved in cell growth and apoptosis, and caspase-3/7 activity were examined. SJWE dose-dependently suppressed cell growth and induced apoptosis of MCF-7 cells. Mechanistically, SJWE enhanced the phosphorylation of AMP-activated protein kinase (AMPK) and decreased the expression of p-mammalian target of rapamycin (p-mTOR) and p-eukaryotic translation initiation factor 4E (eIF4E)-binding protein 1 (4E-BP1). Also, SJWE inhibited the phosphorylation of protein kinase B (Akt) and showed increases in the expression of pro-apoptotic proteins Bax and Bad with decreases in the expression of anti-apoptotic proteins including B-cell lymphoma 2 (Bcl-2), B-cell lymphoma-extra large (Bcl-xL), and p-Bcl-2-associated death promoter (p-Bad). SJWE at 50 μg/mL showed markedly enhanced caspase-7 activation. Taken together, our results provide evidence that SJWE shows anti-proliferative and pro-apoptotic effects via inhibition of AMPK/mTOR and activation of a mitochondrial pathway. Therefore, SJWE can be used as a chemo-preventive agent without photo-activation.

## 1. Introduction

Breast cancer is the most frequently diagnosed cancer among women; it is difficult to be treated when metastasis occurs to bone or brain [1]. The current medical care options of breast cancer include surgery with adjunct radiotherapy, hormonal therapy, chemotherapy and/or targeted biologic therapy [2]. Even with the availability of these current therapies, because of their detrimental side effects, the search for alternative therapies mining the space of natural compounds still continues [3,4].

Severe disturbance, caused by too much growth and too little death, might eventually lead to cancer [5]. Since growth inhibition is the primary action of cancer therapies such as chemotherapy and irradiation, defects in regulation of cell proliferation and/or the apoptotic pathways can make cancer cells resistant to these therapies. Therefore, an obvious strategy for cancer therapy targeting the tumor mass and stopping its metastasis is growth inhibition via suppression of cell proliferation and induction of apoptosis [5]. Recently, there has been renewed interest in AMPK as a new molecular target in the prevention and treatment of cancer by phytochemicals [6,7]. AMPK essentially inhibits all anabolic pathways that promote cell growth [8]. Therefore, considering rapid growth of tumor cells, which requires a tremendous demand for energy, it is not astonishing that AMPK represses the growth of cancer cells. Furthermore, AMPK controls the mTOR which is the major growth regulatory pathway [9]; the mTOR protein comprises two apparent multi-subunit complexes, mTORC1 and mTORC2. The mTORC1 controls growth, proliferation, and survival of cells, and activates the kinases p70S6K1 (S6K1) and 4E-BP1which are involved in mRNA translation. The mTORC2 is resistant to rapamycin inhibitory activity and activates protein kinase C-∝ (PKC-∝) and Akt [9,10]. It is common to defects in the mTOR signaling pathway including phosphoinositide 3-kinase (PI3K) amplification, Akt overexpression and S6K1, 4E-BP1, and elF4E overexpression in cancer [10,11]. Therefore, alterations in components of the mTOR pathway have a major role in tumor progression. The mTORC2 makes the relationship between Akt and mTOR complicated, because Akt and mTOR are linked to each other via positive and negative regulatory circuits, thereby restraining their simultaneous hyper-activation [12]. In addition, it is well known that AMPK is associated with the PI3K-Akt pathway which can activate the mTOR pathway [13]. Akt is a serine threonine kinase which controls critical cellular survival and metabolic processes such as cell cycle and apoptosis [14,15,16,17]. Activation of Akt can initiate apoptosis through the mitochondrial (intrinsic) pathway.

St. John’s Wort (SJW: *Hypericum perforatum*) is an herbaceous plant used for treatment of fibrosis, neuralgia, depression, and anxiety as an alternative to classic antidepressants [17,18,19]. Bioactive compounds of SJW are hypericin, hyperforin, and flavonoids such as flavonol, flavones, bioflavonoids and tannins [20]. Generally, concentrations of hypericin, hyperforin, and rutin have been reported as 0.1–0.15%, 2.0–4.5%, and 1.6%, respectively [20]. Hypericin has been used as a photosensitizer in photodynamic therapy (PDT) with powerful in vivo and in vitro anticancer activity in conjunction with irradiation. The mechanisms eliciting cell death seem to be dependent on cell type and the photodynamic conditions used [21]. In addition, the drug interaction of SJW with anticancer drugs was reported. SJW modulates the expression of multidrug resistance-1 (MDR-1) which is a major mechanism of multidrug resistance responsible for the failure of chemotherapy [20,22,23,24,25].

Recent studies suggest SJW and its active compounds are estrogenic agents to treat postmenopausal syndrome [26,27,28,29]. However, the benefits provided as a phytoestrogen should not be associated with an increased risk of estrogen-sensitive breast, ovarian, and endometrial cancers [30]. In the present study, we investigated the anti-proliferative and pro-apoptotic effects of SJWE to demonstrate the anti-estrogenic and chemo-preventive effects and to explore the mechanism of action in MCF-7 human breast cancer cells.

## 2. Results

### 2.1. SJWE Inhibited the Proliferation in MCF-7 Human Breast Cancer Cells

**Figure 1:**
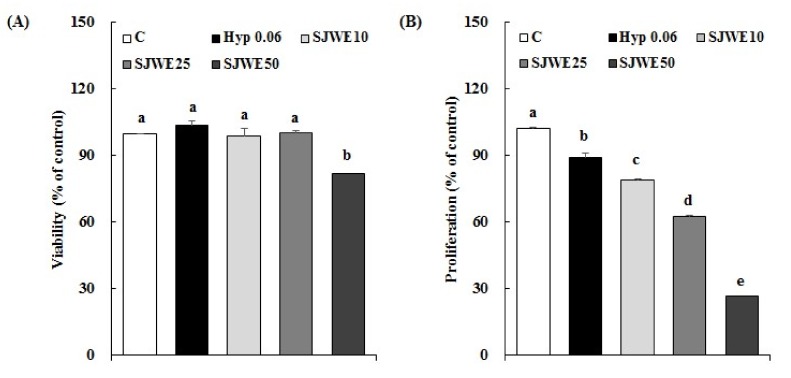
Effect of hypericin and St. John’s Wort extract on viability (**A**) and proliferation (**B**) in MCF-7 cells. MCF-7 cells were treated with DMSO, hypericin, or SJWE (**A**) for 24 h and (**B**) for 5 days (*n* = 9). C: DMSO, Hyp 0.06: hypericin 0.06 μM, SJWE10: 70% ethanol extract of St. John’s Wort 10 μg/mL, SJWE25: 70% ethanol extract of St. John’s Wort 25 μg/mL, SJWE50: 70% ethanol extract of St. John’s Wort 50 μg/mL. Means with the same letter are not significantly different by Duncan’s multiple-range test (*p* < 0.05).

MCF-7 cells were cultured in the absence or presence of SJWE or hypericin, the active compound of SJW, and then viability and proliferation of the cells were examined. Both hypericin and SJWE showed no apparent toxicity in MCF-7 cells treated initially for 24 h (Figure 1A). There was a dose-dependent decrease in proliferation of cells treated with SJWE for 5 days. We compared the growth inhibitory effect of hypericin with that of SJWE in MCF-7 cells treated with hypericin or SJWE for 5 days without photo-activation. The growth inhibition of hypericin was negligible and much lower than that of SJWE (Figure 1B).

### 2.2. SJWE Induced Apoptosis in MCF-7 Human Breast Cancer Cells

**Figure 2:**
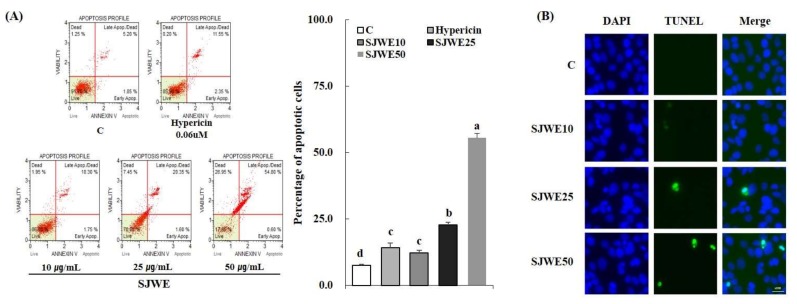
Effect of hypericin and St. John’s Wort extract on apoptotic profile of MCF-7 cells. MCF-7 cells were treated with DMSO, hypericin, or SJWE for 24 h. Apoptotic cells were measured by Annexin V and Dead cell kit (**A**) and TUNEL assay (**B**). C: DMSO, Hyp 0.06: hypericin 0.06 μM, SJWE10: 70% ethanol extract of St. John’s Wort 10 μg/mL, SJWE25: 70% ethanol extract of St. John’s Wort 25 μg/mL, SJWE50: 70% ethanol extract of St. John’s Wort 50 μg/mL. Means with the same letter are not significantly different by Duncan’s multiple-range test (*p* < 0.05).

SJWE dose-dependently increased apoptosis of MCF-7 cells treated for 24 h. Cells in the lower-right quadrant (cells in the early stages of apoptosis: Annexin V-PE(+) and Dead Cell Marker(−)) and in the upper-right quadrant (cells in the late stages of apoptosis or dead by apoptotic mechanism: Annexin V-PE(+) and Dead Cell Marker(+)) were dose-dependently increased by SJWE (Figure 2A). Since the effect of hypericin on cell growth and apoptosis was negligible without photo-activation, MCF-7 cells were treated with SJWE only for the rest of experiment. Furthermore, the apoptotic morphology alteration in MCF-7 cells was detected by terminal deoxynucleotidyl transferase dUTP nick end labeling (TUNEL) assays. The presence of TUNEL-positive cells with fragmented DNA was indicated by a green fluorescence signal, indicating that DNA strand breaks had occurred. SJWE increased TUNEL-positive cells in MCF-7 cells (Figure 2B).

### 2.3. AMPK/mTOR/4E-BP1 Pathway Was Involved in SJWE Induced Growth Inhibition of MCF-7 Human Breast Cancer Cells

**Figure 3:**
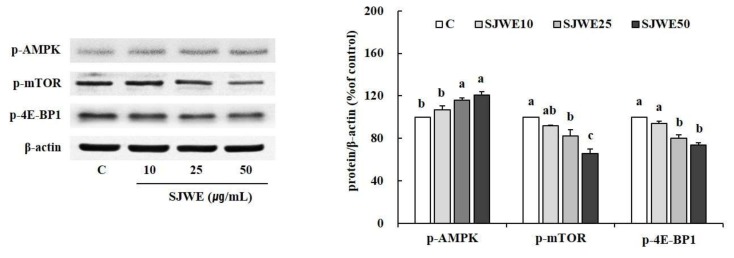
Effect of St. John’s Wort extract on mTOR pathway protein expression in MCF-7 cells. MCF-7 cells were treated with 70% ethanol extract of St. John’s Wort (SJWE 10, 25 or 50 μg/mL) for 6 h. The expression of mTOR pathway proteins was detected by Western blotting analysis and protein was quantified by Vision Works image analysis software (UVP). β-actin served as a loading control. C: DMSO, SJWE10: 70% ethanol extract of St. John’s Wort 10 μg/mL, SJWE25: 70% ethanol extract of St. John’s Wort 25 μg/mL, SJWE50: 70% ethanol extract of St. John’s Wort 50 μg/mL. Means with the same letter are not significantly different by Duncan’s multiple range test (*p* < 0.05).

Because p-AMPK, an active form of AMPK is considered as an antigrowth molecule via inhibitory effects on mTOR, we examined the effect of SJWE on the AMPK/mTOR pathway in MCF-7 cells. SJWE dose-dependently increased the protein expression of p-AMPK in MCF-7 cells treated for 6 h (Figure 3). In addition, the expression level of p-mTOR, the downstream of AMPK, and p-4E-BP1, the direct downstream of mTOR, was effectively suppressed by SJWE.

### 2.4. SJWE Caused Hypophosphorylation of Akt in MCF-7 Human Breast Cancer Cells

**Figure 4:**
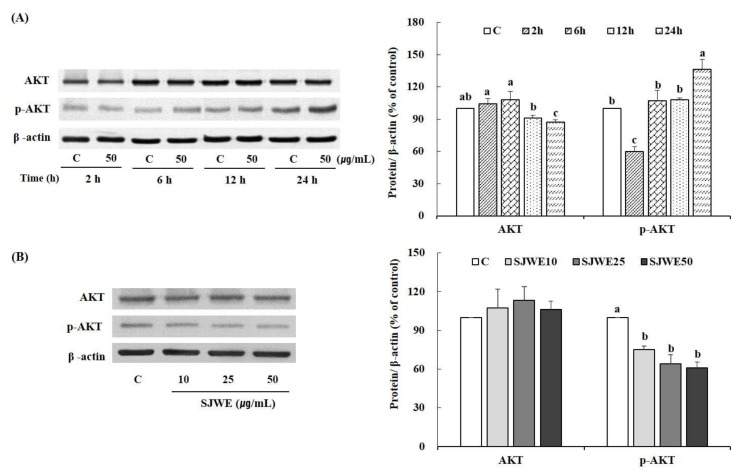
Effect of St. John’s Wort extract on Akt and p-Akt expression in MCF-7 cells. (**A**) MCF-7 cells were treated with DMSO or SJWE for 2, 6, 12, or 24 h; (**B**) MCF-7 cells were treated with DMSO or SJWE for 2 h. The expression of AKT and p-AKT was detected by Western blotting analysis and protein was quantified by Vision Works image analysis software (UVP). β-actin served as a loading control. C: DMSO, SJWE10: 70% ethanol extract of St. John’s Wort 10 μg/mL, SJWE25: 70% ethanol extract of St. John’s Wort 25 μg/mL, SJWE50: 70% ethanol extract of St. John’s Wort 50 μg/mL. Means with the same letter are not significantly different by Duncan’s multiple range test (*p* < 0.05).

We examined SJWE-induced hypophosphorylation of Akt. MCF-7 cells were treated with 50 μg/mL of SJWE for 2, 6, 12 or 24 h. As shown in Figure 4A, 50 μg/mL of SJWE inhibited Akt phosphorylation at serine 473 relative to control cells after a brief, 2 h, exposure with partial recovery after 6 h. We also determined the Akt phosphorylation in MCF-7 cells treated with various concentrations of SJWE for 2 h. SJWE treatment led to no differences in the expression of Akt but there was a decrease in the expression of p-Akt following a 2 h exposure (Figure 4B). This result suggests that SJWE treatment for a short time inhibited this important signaling pathway in breast cancer cells.

### 2.5. SJWE Induced Apoptosis Was Mitochondrial in Origin and Was Regulated by the Bcl-2 Family in MCF-7 Human Breast Cancer Cells

**Figure 5:**
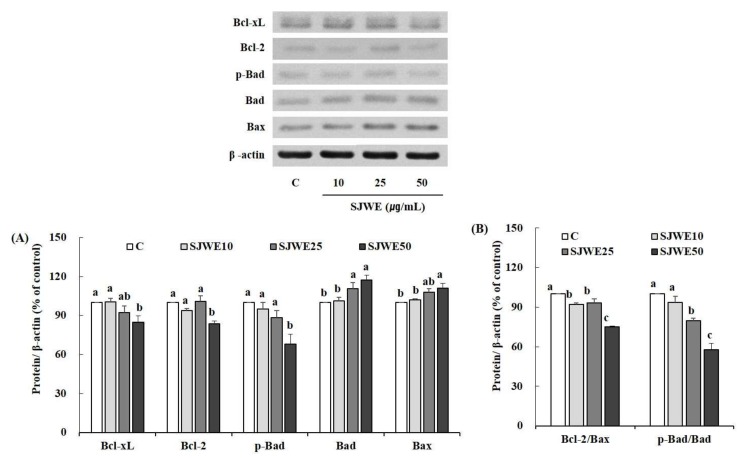
Effect of St. John’s Wort ethanol extract on Bcl-2 family protein expression in MCF-7 cells. MCF-7 cells were treated with DMSO or SJWE for 6 h to measure the expression of Bcl-2 family proteins using Western blotting. Protein was quantified by Vision Works image analysis software (UVP). β-actin served as a loading control. (**A**) Bcl-2 family; (**B**) Bal-2/Bax and p-Bad/Bad ratio. C: DMSO, SJWE10: 70% ethanol extract of St. John’s Wort 10 μg/mL, SJWE25: 70% ethanol extract of St. John’s Wort 25 μg/mL, SJWE50: 70% ethanol extract of St. John’s Wort 50 μg/mL. Means with the same letter are not significantly different by Duncan’s multiple range test (*p* < 0.05).

We examined the effect of SJWE on the expression of Bcl-2 family members: anti-apoptotic (Bcl-2 and Bcl-xL) and pro-apoptotic proteins (Bax and Bad). We determined the expression of Bcl-2 family proteins in the MCF-7 cells treated with various concentration of SJWE for 6 h. The expression of anti-apoptotic proteins, Bcl-xL, Bcl-2, and p-Bad, was reduced, whereas the expression of pro-apoptotic proteins, Bad and Bax was increased by higher concentrations of SJWE (Figure 5A). SJWE showed dose-dependent decreases in the expression ratios of Bcl-2/Bax and p-Bad/Bad (Figure 5B).

### 2.6. SJWE Increased Caspase-7 Activation in MCF-7 Human Breast Cancer Cells

**Figure 6:**
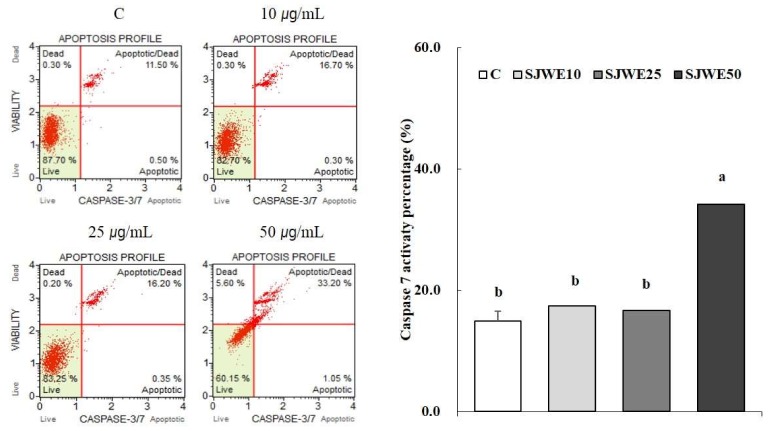
Effect of St. John’s Wort ethanol extract on caspase 7 in MCF-7 cells. MCF-7 cells were treated with DMSO or SJWE for 24 h and analyzed using Muse^TM^ caspase-3/7 kit. C: DMSO, SJWE10: 70% ethanol extract of St. John’s Wort 10 μg/mL, SJWE25: 70% ethanol extract of St. John’s Wort 25 μg/mL, SJWE50: 70% ethanol extract of St. John’s Wort 50 μg/mL. Means with the same letter are not significantly different by Duncan’s multiple range test (*p* < 0.05).

We measured the activity of caspase-7 to demonstrate that SJWE induced activation of caspase-7 to propagate apoptosis of MCF-7 cells. As shown in Figure 6, 50 μg/mL SJWE increased caspase-7 activation in MCF-7 cells.

## 3. Discussion

SJWE showed no toxicity at up to a concentration of 50 μg/mL following 24 h of exposure. However, a 5-day exposure of SJWE inhibited cell proliferation at the same concentration (Figure 1). In order to compare the anti-proliferative effect of hypericin alone with that of SJWE, we determined the cytotoxicity and proliferation in MCF-7 cells treated with 0.06 μM hypericin the same concentration as 30 μg/mL SJWE. Hypericin, without photo-activation, showed a negligible effect on the proliferation of MCF-7 cells (Figure 1B). Hypericin has been tested for experimental photodynamic therapy of cancer [21,31,32,33]. To demonstrate the induction of the apoptosis process involved in the suppressive effect of SJWE on the growth of MCF-7 cells, we assessed apoptosis in MCF-7 cells with the Muse^TM^ Annexin V and Dead Cell Kit. SJWE dose-dependently induced apoptosis of MCF-7 cells. As expected, hypericin without photo-activation never had a really wide influence on the apoptosis of MCF-7 cells (Figure 2A). Mirmalek et al. [34] reported that MCF-7 cells treated with 5 μg/mL (10 μM) hypericin showed similar apoptosis rates with 50 μg/mL of SJWE. Moreover, TUNEL-positive cells were increased in SJWE treatment (Figure 2B).

Next, in order to characterize the underlying mechanism of SJWE-induced anti-proliferative effect, we examined the effect of SJWE on AMPK/mTOR/4E-BP1 signaling in MCF-7 cells. Cell growth requires availability of energy and balanced nutrients. In this regard, AMPK prevents cell growth by inhibiting energy production for synthesis of rRNA, protein, and lipids. Furthermore, AMPK is part of a tumor suppressor network that regulates cell growth and proliferation [35]. In the present study, SJWE showed a dose-dependent increase in the protein expression of p-AMPK (Figure 3). In general, mTORC1 regulates cell growth in response to nutrient availability and growth factors [10]. The mTORC1 and its direct downstream protein, 4E-BP1 regulate several cellular functions that are critical to tumorigenesis such as cellular proliferation, growth, survival, and mobility [36,37]. mTORC1 phosphorylates 4E-BP1 at multiple sites and promotes the dissociation of elF4E from 4E-BP1, reducing the inhibitory effect of 4E-BP1 on the initiation of eIF4E-dependent translation [10,38]. As expected, the protein expression of p-mTOR and p-4E-BP1 was effectively reduced by SJWE treatment. These results suggest that SJWE phosphorylates AMPK thereby inhibiting the phosphorylation of mTOR and 4E-BP1, resulting in decreased proliferation of MCF-7 cells.

Defects in specific pathways for apoptosis may enable survival of tumor cells under conditions that would have otherwise led to their death [39]. Two alternative pathways mediate apoptosis in various cell types. These are intrinsic and extrinsic pathways mediated by mitochondria and death receptors on the cell surface, respectively [40]. Caspases, a family of cysteine proteases, are activated in both pathways [41,42] and lead to distinct biochemical and morphological characteristics such as cell shrinkage, chromatin condensation, and DNA fragmentation [42,43]. MCF-7 cells are one of the most resistant cells to treatment with PDT activated hypericin as they have relatively high levels of Bcl-2, an anti-apoptotic protein [44]. Moreover, the mTORC2 is known to phosphorylate Akt at Ser473, which plays an important role in apoptosis [10]. Therefore, we have studied the effect of SJWE on the Akt phosphorylation at Ser473 and apoptosis signaling through mitochondria. Akt plays an important role in the growth and survival of many types of cancer cells, including breast carcinoma cells [45,46]. The phosphorylation of Akt inhibits the expression of Bad, a pro-apoptotic protein [40]. The results of the present study showed that SJWE inhibited the phosphorylation of Akt in MCF-7 cells following 2 h of exposure, but the recovery of the Akt phosphorylation was observed after 6 h. The partial recovery of Akt phosphorylation after a brief exposure was also observed in another study. Giannelli et al. [47] revealed that LY294002, the inhibitor of PI3K/Akt, completely inhibited the expression of p-Akt after 6 h of exposure with partial recovery at 24 h. Furthermore, after a 24-h exposure, LY294002 inhibited matrix metalloproteinase (MMP)-2 and -9 secretions that are downstream of the Akt pathway. In the present study, Bad and caspase, the pro-apoptotic proteins, remained inhibited after 6 and 24 h of exposure to SJWE even after the recovery of Akt phosphorylation. Based on this result, it is reasonable to speculate that hypophosphorylation of Akt, in part, accounts for the anti-proliferative effect of SJWE. As mentioned above, clinical trials of mTOR inhibitors could result in Akt hyperactivation. This is one problem associated with therapeutic approaches using rapamycin which blocks some actions of mTOR but not all, thereby consequently activating regulatory feedback loops involving the mTORC2 complex [10,12]. However, in the present study, SJEW showed inhibitory effects on proliferation and apoptosis of MCF-7 cells via inhibiting mTOR/Akt^Ser473^ as well as mTOR/4E-BP1.

In the present study, SJWE showed increases in the expression of pro-apoptotic proteins including Bax and Bad, with concomitant decreases in the expression of anti-apoptotic proteins including Bcl-2, Bcl-xL, and p-Bad. Bcl-xL, not Bcl-2, is demonstrated to sequester Bak, a pro-apoptotic protein [48]. Bad is known to be involved in the displacement of Bak from its interaction with Bcl-xL, and dephosphorylation of Bad is required for reducing Bak/Bcl-xL interaction [49]. In the present study, SJWE induced dephosphorylation of Bad and, moreover, showed a decrease in the expression of Bcl-xL protein, which is expected to free Bak from Bak/Bcl-xL sequestering. A previous study demonstrated that dephosphorylation of Bad at serine 75 by LY294002 is most likely a consequence of the inhibition of Akt activity [47]. From our results, we can assume that SJWE inhibits Akt activity leading to dephosphorylation of Bad and freeing Bak from a Bak/Bcl-xL complex. Freed Bak led to apoptosis induction. We also observed that SJWE increased the expression of Bax and decreased the expression of Bcl-2, thereby decreasing the ratio of Bcl-2 to Bax.

Besides Bcl-2 family members, a family of cysteine proteases plays an important role in regulating apoptosis [40,50]. MCF-7 cells lack caspase-3; thereby caspase-7 may take over deficits in caspase-3 function for mitochondrial-dependent apoptosis. In the present study, we found markedly enhanced caspase-7 activation by 50 μg/mL of SJWE. Akt suppresses the release of cytochrome c and inactivation of caspase-9, thereby promoting cell survival [51]. The activation of caspase-9 resulted in the activation of caspases-3 and -6, starting the chain of events leading to apoptosis [50]. Taken together, SJWE induced apoptosis of MCF-7 cells via hypophosphorylation of Akt, decreased the expression of pro-apoptotic proteins and increased the expression of anti-apoptotic proteins.

In the present study, we provide evidence that SJWE suppresses cell proliferation and induces apoptosis in MCF-7 human breast cancer cells through AMPK/mTOR and apoptotic signaling through the mitochondria. This growth inhibitory effect of SJWE is not related to photo-activation, because we used the extract of SJWE without irradiation and demonstrated a negligible effect of hypericin in the absence of irradiation. Ferenc et al. [33] demonstrated that a combination of PDT with hypericin and genistein is significantly effective in the reduction of proliferation as well as induction of apoptosis in human breast cancer cells. Hyperforin, another bioactive compound, was reported to inhibit tumor cell proliferation via induction of apoptosis by triggering activation of the mitochondrial pathway and caspases in the absence of photo-activation [52]. In the present study, in addition to the mitochondrial pathway and caspases, we demonstrated that the AMPK/mTOR/4E-BP1 pathway was involved in SJWE-induced growth inhibition of MCF-7 human breast cancer cells. Moreover, previous studies also revealed that SJW down-regulated MDR-1 expression, a major mechanism of multidrug resistance, by components other than hypericin such as hyperforin, flavonol, flavones, biflavonoids, tannins, and others [20,25]. Therefore, the anti-proliferative and pro-apoptotic effects of SJWE may be a combination of its ingredients including hypericin, hyperforin, and flavonoids. Based on our results, we can conclude that SJWE, without photo-activation, can be used as a chemo-preventive agent via growth inhibition of human breast cancer cells.

## 4. Materials and Methods

### 4.1. Preparation of St. John’s Wort Extract

St. John’s Wort (SJW) were cultivated in China and purchased from Anhui Yiyuan Biotechnology Co., Ltd. (Yiyuan, China). SJW extracts were prepared using the protocol previously established [29]. In brief, SJW were soaked in 5 L of 70% ethanol at 90 °C for 8~12 h, and this procedure was repeated twice. The St. John’s Wort ethanol extract (SJWE) were filtered, evaporated, and freeze-dried. The final extraction yield was 16.3%. The freeze-dried powder obtained were then dissolved in DMSO and stored at −20 °C for further use.

### 4.2. Cell Culture and Treatment

MCF-7 cells were purchased from the American Type Culture and Collection (ATCC, Rockville, MD, USA). The cells were cultured in in Dulbecco’s modified eagle’s medium (DMEM, Gibco, Gaithersburg, MD, USA) containing 10% fetal bovine serum (FBS) and 1% penicillin/streptomycin and incubated in a humidified atmosphere of 5% CO_2_ at 37 °C. The cells were incubated with various concentrations of SJWE (10~50 μg/mL). For comparison with the effect of hypericin, cells were treated with hypericin 0.06 μM. The concentration of 0.06 μM hypericin was chosen for treatment based on the medium concentration of 30 μg/mL of SJWE. The hypericin in SJWE totaled 0.06 mg/g according to our preliminary studies.

### 4.3. Cell Proliferation

Cells were plated at 6 × 10^4^/well in 6-well culture plates and incubated overnight to allow attachment to occur. Subsequently, the cells were treated with DMSO, hypericin or SJWE. This media were replaced every other day. After treatment for 5 days, cells were washed twice with PBS and then counted. Result was presented as relative percentage to the control of each group.

### 4.4. Apoptosis

For the assessments of apoptosis, the Muse^TM^ Annexin V and Dead Cell Kit was used (Merck KGaA, Darmstadt, Germany). MCF-7 cells were incubated with either DMSO, SJWE or hypericin for 24 h. The treatment medium was then removed and the cells were washed with PBS and were collected using trypsin-EDTA. The harvested cells were centrifuged at 1400 rpm for 5 min and re-suspended with a complete medium. Muse^TM^ Annexin V and Dead Cell reagent (100 μL) was added to each of the re-suspended cell and incubated for 20 min. The re-suspended cells were assayed by Muse^TM^ Cell Analyzer.

### 4.5. TUNEL Assay

The apoptotic cells were also detected using DeadEnd^TM^ Fluorometric TUNEL System (Promega Corp., Madison, WI, USA) according to the manufacturer’s instructions. Briefly, MCF-7 cells in chamber slides were treated with DMSO or SJWE for 24 h, fixed with 4% paraformaldehyde and permeabilized in 0.1% triton X-100. The slides were incubated with TUNEL reaction mixture and analyzed under a fluorescence microscope (original magnification, ×200).

### 4.6. Immunoblotting

MCF-7 cells were washed once with cold PBS and scraped into lysis buffer (10 mM Tris-HCl (pH 7.4), 100 mM NaCl, 1 mM EDTA, 1 mM EGTA, 1 mM NaF, 20 mM Na_4_P_2_O_7_, 2 mM Na_3_VO_4_, 1% Triton-X 100, 10% glycerol, 0.1% sodium dodecyl sulfate (SDS), 0.5% deoxycholate, 1 mM PMSF, 5% protease inhibitor cocktail) for 10 min. The lysis mixture was clarified at 13,000 rpm for 20 min at 4 °C. The supernatant was collected as the lysate, and the protein concentration was determined by a Bradford protein assay (Sigma, St. Louis, MO, USA). The 25 μg of lysate protein was mixed with 5× SDS-PAGE sample buffer containing 60 mM Tris-HCl (pH 6.8), 25% glycerol, 2% SDS, 10% bromophenol, and 5% 2-mercaptoethanol, and heated at 95 °C for 10 min, and separated by SDS-PAGE. After electrophoresis, the proteins were transferred onto a nitrocellulose membrane using a transblot chamber with Tris transfer buffer (0.025 M Tris-HCl, 0.192 M glycine, and 20% MeOH). The membrane was blocked for 1 h at 4 °C with 5% nonfat milk in TBS-T buffer containing 10 mM Tris-HCl (pH 6.8), 100 mM NaCl, and 0.1% Tween 20. The membranes were then incubated overnight with either of the following primary antibodies from Cell Signaling Technology (Beverly, MA, USA): p-AMPK, p-mTOR, p-4E-BP1, Akt, p-Akt, Bax, Bcl-2, Bad, p-Bad, and Bcl-xL (all used at 1:1000 dilution). In addition, antibody from β-actin was from Santa Cruz Biotechnology (Santa Cruz, CA, USA) (also used 1:1000). After washing with TBS-T buffer, the membranes were incubated with horseradish peroxidase conjugated secondary antibody (goat anti-rabbit or goat anti-mouse at 1:2000) for 1 h at room temperature. Bands were detected by chemiluminescent substrate (IMGENEX, San Diego, CA, USA), and quantified using UVP imaging system (UVP, Upland, CA, USA) and Vision Works image analysis software (UVP).

### 4.7. Caspase 7 Activation

The assay was performed using Muse^TM^ caspase-3/7 kit, according to the manufacturer’s protocol (Merck KGaA, Darmstadt, Germany). Briefly, MCF-7 cells (1 × 10^6^ cells) were seeded in 6-well plates and incubated with SJWE (or DMSO) for 24 h. The cells were then trypsinized and washed with PBS. The cell samples were prepared in 1xAssay buffer (provide with the kit). Five micro-liters of caspase-3/7 working solution was added to 50 μL of cells and incubated at 37 °C for 30 min. Next 150 μL of 7-aminoactinomycin D (AAD) working solution was added to the cells, mixed thoroughly and ran on the Muse^TM^ Cell Analyzer (Merck KGaA) for flow cytometry. The data was obtained from 5000 events (gated cells) per sample. The percentages of positive cells were defined as exceeding the mean fluorescence intensity of top 10% of the control cells.

### 4.8. Statistical Analysis

Statistics were analyzed using SPSS version 23.0 (SPSS Inc, Chicago, IL, USA). The results were expressed as the mean ± S.E. of three independent experiments. Moreover, the comparisons were based on a one-way ANOVA, followed by Duncan’s multiple-range test. A *p*-value < 0.05 was considered statistically significant.

## Abbreviations

AMPK	AMP-activated protein kinase
mTOR	mammalian target of rapamycin
S6K1	kinases p70S6K1
eIF4E	eukaryotic translation initiation factor 4E
4E-BP1	eIF4E-Binding protein 1
PKC-∝	protein kinase C-∝
Akt	protein kinase B
Bcl-xL	B-cell lymphoma-extra large
Bcl-2	B-cell lymphoma 2
Bad	Bcl-2-associated death promoter
Bax	Bcl-2 like protein 4
